# Assessing the Feasibility of Mid- and Near-Infrared Spectroscopy for Classifying Plant-Based Beverages and Predicting Nutrient Content

**DOI:** 10.3390/foods15152588

**Published:** 2026-07-23

**Authors:** Alberto Guerra, Massimo De Marchi, Marta Pozza, Carmen L. Manuelian

**Affiliations:** 1 Department of Agronomy, Food, Natural Resources, Animals and Environment (DAFNAE), University of Padova, Viale dell’Università 16, 35020 Legnaro, Italy; alberto.guerra@unipd.it (A.G.); massimo.demarchi@unipd.it (M.D.M.); marta.pozza.2@unipd.it (M.P.); 2 Group of Ruminant Research (G2R), Department of Animal and Food Sciences, Universitat Autònoma de Barcelona (UAB), 08193 Bellaterra, Spain

**Keywords:** compositional analysis, infrared spectroscopy, quality control, plant-based beverages

## Abstract

The increasing demand for plant-based beverages as alternatives to dairy milk requires rapid and reliable methods to assess their composition and authenticity. This study investigated the feasibility of mid-infrared (MIR) and near-infrared (NIR) spectroscopy for classifying plant-based beverages and predicting their nutritional profile. A total of 57 commercial beverages from five categories (oat, almond, soybean, rice, and coconut) were analyzed. Canonical discriminant analysis was used to discriminate among beverage categories, and modified partial least-squares regression models were developed using reference chemical analyses and spectral data to predict protein, fat, sugars, ash, acidity traits, minerals, and amino acid composition. Both MIR and NIR successfully discriminated among the five beverage categories. Quantitative prediction models were generally more accurate with MIR than with NIR. The most robust MIR models were obtained for protein, fat, glucose, ash, and selected minerals (P and K), reaching accuracy levels suitable for quality-control applications. Protein was predicted with excellent accuracy by both technologies. The most robust prediction models were achieved for amino acid composition, with all amino acids except phenylalanine showing satisfactory predictive performance, particularly with MIR spectroscopy. In conclusion, these results demonstrate the feasibility of infrared spectroscopy for the authentication and compositional assessment of plant-based beverages. In particular, MIR spectroscopy showed considerable potential for integration into routine quality-control workflows, similar to those currently implemented for dairy milk analysis.

## 1. Introduction

In Western countries, there has been a rise in plant-based beverage (PBB) consumption as substitutes for milk driven by concerns for human health, environmental impact, and ethical considerations related to milk production [1], along with lactose intolerance, cow’s milk allergy, and dietary choices such as veganism and flexitarianism [2,3,4]. Plant-based beverages are expected to grow at a compound annual rate of 10% from 2023 to 2028, reaching USD 43.6 billion [5]. Those products have a ‘milky’ aspect and are commonly marketed as dairy replacers [6]. The most common PBB are soybean-, almond-, and coconut-based beverages [4], and more recently, oat-based beverages [6]. Thus, many major dairy companies (e.g., Arla Foods, Danone, DMK, Nestlé, Müller, Granarolo) have begun offering PBB to meet consumers’ demands for milk alternatives [7]. Nevertheless, consumers of these products should be aware that PBBs present several nutritional and technological limitations when compared to bovine milk [8]. Many products contain stabilizers, thickeners, or added sweeteners, which may cause gastrointestinal discomfort or contribute to inflammatory responses [8]. Additionally, certain plant-derived ingredients (e.g., nuts, legumes) may induce allergic reactions in susceptible individuals [9], and the proteins present in PBBs generally lack the completeness and high biological value characteristic of dairy milk proteins [10]. Consequently, although PBBs may serve as suitable substitutes in specific circumstances, their broader use as direct nutritional replacements for dairy milk remains a subject of ongoing scientific evaluation.

For milk quality evaluation, particularly fat, protein, and main fatty acid group content, the dairy industry relies on mid-infrared (MIR) [11] and near-infrared (NIR) [12,13] technologies, as these are fast, cost-effective, non-destructive, and environmentally friendly methods. Moreover, AOAC International [14] and the IDF (International Dairy Federation) consider MIR a reference method for determining the content of milk fat, protein, and lactose [6,15]. However, developing reliable prediction models requires high-quality spectrometers and reference analysis, which increases the initial cost during instrument setup. Few studies have developed infrared prediction models for PBBs, primarily focusing on the sugar, protein, and fat content of soybean-based beverages and the detection of PBB adulteration. Moreover, these studies do not compare the accuracy of the two techniques, MIR and NIR, and focus only just on one of them. Regarding MIR spectra, excellent prediction models have been reported on soybean-based beverages for total sugars [16,17], and screening prediction models have been developed for total protein [16]. The MIR spectra have also been described in coconut-based beverages [18], and prediction models have only been developed to accurately detect product adulteration with water [19]. A recent study applied principal component analysis (PCA) and hierarchical cluster analysis to MIR spectra of four PBBs (almond, rice, oat, and soybean) and clustered rice, oat, and soybean-based beverages, but almond-based beverages were not clearly separated [20]. Regarding NIR spectra, excellent models have been developed to predict moisture, fat, protein, and pH in grated coconut (raw and defatted) samples from coconut-based beverages [21]. Moreover, benchtop and portable NIR devices have been applied to accurately detect coconut-based beverage adulterer with corn flour and tapioca [22]. Good prediction models for detecting adulteration with soybean, starch, polydextrose, and maltodextrin in rice-based beverages have also been described [23].

There is an interest in applying the same technology in PBBs to determine its composition as it is already used for milk. To our knowledge, infrared spectra have only been comprehensively characterized for soybean-, coconut-, almond-, and rice-based beverages. Moreover, none of the available studies on the topic compared the performance of both technologies, included more than one PBB to develop the prediction models, or covered a detailed profile of fatty acids, minerals, and amino acids. Thus, this study investigated the feasibility of MIR and NIR spectroscopy to discriminate among the most common PBBs (i.e., oats, almonds, soybeans, rice, and coconuts) and to predict their gross composition, acidity traits, and detailed composition (fatty acid profile and content, amino acid content, and mineral content) using a global calibration developed across PBB categories.

## 2. Materials and Methods

### 2.1. Sample Collection

A total of 57 commercial units (brick cartons) of 5 different PBB categories were purchased (oat, *n* = 12; almond, *n* = 12; soybean, *n* = 12; rice, *n* = 11; and coconut, *n* = 10; Table S1). The samples were transported to the Department of Agronomy, Food, Natural resources, Animals and Environment (DAFNAE) of the University of Padova (Legnaro, Italy) and stored under controlled conditions until analysis, which was completed within two days of purchase. On the day of the analysis, each carton was thoroughly homogenized and divided into several 10 mL aliquots to ensure representativeness during spectral acquisition and reference analyses.

The selected PBB varieties represent the most widely consumed non-fortified ultra-high-temperature (UHT) formulations. All samples were purchased in northeastern Italy at different supermarkets during the first semester of 2022. The sampling was strategically conducted over two separate days and involved a wide diversity of international brands and production batches to maximize the captured variability. Although purchased locally, the brands included are major players in the European Union market, with production sites and distribution networks across multiple EU countries (e.g., Belgium, Germany, and Spain), thus ensuring that the dataset reflects a broad commercial landscape [8]. Such variability in chemical and physical traits is essential for the development of robust and transferable calibration models [24], as it allows the equations to account for the inherent diversity of industrial formulations (Table 1 and Table 2).

### 2.2. Spectral Collection

For each sample, MIR and visible-NIR spectra were collected after homogenization by warming the sample at 38 °C for 30 min in a water bath and gently inverting it. Both instruments worked at room temperature (25 °C). Following the manufacturer recommendation, MIR spectra was obtained using a Milkoscan FT3 (FOSS Electric A/S; Hillerød, Denmark), which provided a total of 1060 data points per spectrum, ranging from 5000 to 900 cm^−1^. Absorbance was recorded as log(1/transmittance). Visible-NIR spectra were collected with a DS2500 (FOSS Electric A/S; Hillerød, Denmark), which works from 400 nm to 2500 nm with a spectral resolution of 0.5 nm. Each visible-NIR spectrum represented an average of 32 sub-spectra collected via automatic rotation of the slurry sample cup (40 mm diameter) of FOSS (Electric A/S; Hillerød, Denmark), and absorbance was recorded as log(1/reflectance).

### 2.3. Chemical Analysis

To determine fat (%), protein (%), lactose (%), fructose (%), glucose (%), ashes (%), pH, titratable acidity (°SH; degrees Soxhlet–Henkel), fatty acid profile (% and g/100 g beverage), amino acid content (mg/100 g beverage), and mineral content (Ca, P, Mg, K, Na, and Zn as mg/kg beverage; I as µg/kg beverage), samples were initially freeze-dried. All wet chemistry analyses were performed in duplicate at DAFNAE, except for I content, which was analyzed at Eurolab (Bassano, Italy).

Briefly, ash content was determined by incinerating 5 g of DM at 550 °C (#945.46; AOAC [14]). Protein was quantified using the Kjeldahl method (#991.20; AOAC [14]). Lipids were extracted after hydrolysis with 4 M HCl using petroleum ether: diethyl ether (50:50 *v*/*v*) and a SOXTECH 255 FOSS TECTOR system (FOSS Electric A/S; Hillerød, Denmark). Fructose and glucose were extracted with 0.1 N H_2_SO_4_, lactose with Carrez I and II salts (Merck; Darmstadt, Germany), and quantified by high-performance liquid chromatography (HPLC; Jasco Corporation, Tokyo, Japan) using an Aminex HPX-87H column (300 mm × 7.8 mm; Bio-Rad; Hercules, CA, USA) at 65 °C with 0.0025 N H_2_SO_4_ as the mobile phase (0.6 mL/min). The pH and titratable acidity were measured using an EcoTitrator system (Methohm AG; Herisau, Switzerland).

Fatty acids were extracted with hexane:isopropanol (3:2 *v*/*v*) at 110 °C (3 cycles), esterified overnight at 50 °C with methanol + 1% H_2_SO_4_, and analyzed using a gas chromatograph (GC Agilent 7820; Agilent Technologies; Santa Clara, CA, USA) with an Omegawax column (24136 Supelco; Sigma-Aldrich, Castle Hill, Australia); H_2_ was the carrier gas). Fatty acids were expressed as % of total and g/100 g beverage using INRAN [25] conversion factors: oat, 0.940; almond, 0.956; soybean, 0.800; rice, 0.850; and coconut, 0.942.

Minerals (Ca, calcium; P, phosphorus; Mg, magnesium; K, potassium; Na, sodium; and Zn, zinc) were determined via ICP-OES after microwave digestion with HNO_3_ and H_2_O_2_ [26]. Accuracy was verified using blanks and control solutions. Iodine (I) was determined via ICP-MS after ammonia treatment and heating [27].

Amino acids (histidine, arginine, serine, glycine, aspartic acid, glutamic acid, threonine, alanine, proline, lysine, methionine, tyrosine, valine, cysteine, isoleucine, leucine, and phenylalanine) were analyzed after hydrolysis and pre-column derivatization using high-performance liquid chromatography (Agilent 1260 Infinity HPLC, C18 column, 45 °C; Agilent Technologies; Santa Clara, CA, USA). Tryptophan was determined in accordance with EC Directive 2000/45/EC [28] using basic hydrolysis. Quantification used the AccQ-Tag Ultra Derivatization Kit according to the manufacturer’s instructions (Waters Corporation, Milford, MA, USA).

### 2.4. Statistical Analysis and Development of Prediction Models

Descriptive statistics were computed using R version 4.3.3 [29] with the ‘stats’ package. Spectral data classification by sample category was analyzed using SAS software (version 9.4, SAS Institute Inc., Cary, NC, USA). To reduce dimensionality and identify the most discriminative wavelength among sample categories, a stepwise discriminant analysis was performed using PROC STEPDISC. Variables were entered or removed based on Wilks’ Lambda, using significance levels of 0.01 for entry and stay. The subset of selected variables was then used in a canonical discriminant analysis (CDA) through PROC CANDISC to derive canonical functions summarizing between-group variation. Eigenvalues, canonical correlations, and the percentage of variation explained by each canonical function were computed to assess discrimination power. Subsequently, linear discriminant classification was performed using PROC DISCRIM, assuming equal covariance matrices across groups. Posterior probabilities and misclassification rates were computed via cross-validation to assess classification accuracy. Finally, visualization of group separation was achieved by plotting the 1st and 2nd canonical scores using PROC SGPLOT. Scatter plots were generated with observations colored by sample categories, and axis labels included the percentage of between-group variation explained by each canonical function.

Chemometric analysis for quantitative prediction models was performed using WinISI 4 software (Infrasoft International; Port Matilda, PA, USA). Prediction models for fatty acid content were not developed due to their very low content, as can be observed in Table 2. The amount of the trait on which a prediction model is developed is essential for achieving good calibration models, as absorbance depends on the number of molecular bonds that can be excited [30,31]. Despite this being the first study to attempt to develop prediction models for the fatty acid profile of PBB, to the best of our knowledge, only a previous study on oat flakes and cakes was able to create accurate prediction models for C18:2 (R^2^c = 0.93) and C16:0 (R^2^c = 0.92; [32]).

Predictive equations for all the other traits were developed by a modified partial-least squares algorithm [33]. Model performance was evaluated based on 5-fold cross-validation. This internal validation strategy was selected to maximize the information extracted from the available dataset while ensuring a reliable estimation of the prediction error. The cross-validation approach has been shown to provide prediction models with performance metrics highly aligned with those obtained through an external validation approach in similar food matrices [34]. Therefore, it represents a scientifically sound methodology for evaluating the potential of infrared spectroscopy when dealing with high-cost analytical profiles and finite sample sizes. To improve calibration accuracy, spectral outliers were identified and removed using the Mahalanobis distance (Global H > 3.0), followed by three iterations of eliminating chemical outliers using the T-statistic (>3.0) following the manufacturer recommendation. The scatter corrections applied to the spectra were no correction, detrending (D), standard normal variate (SNV), SNV and D (SNV + D), and multiplicative scatter correction combined with 4 derivative mathematical treatments (0,0,1,1; 1,4,4,1; 1,8,8,1; 2,5,5,1). In the mathematical treatments, the first digit represents the derivative number, the second digit denotes the gap over which the derivative is computed, the third digit refers to the number of data points in the first smoothing, and the fourth digit indicates the number of data points in the second smoothing. The optimal calibration models were identified by selecting the number of latent factors (LFs) that minimized the root-mean-square error of cross-validation, achieved the lowest standard error of cross-validation (SE_CV_), maximized the coefficient of determination in calibration (R^2^_C_) and cross-validation (R^2^_CV_) and resulting in a high residual predictive deviation (RPD), calculated as the ratio between standard deviation and SE_CV_. The interpretation of R^2^_CV_ values followed the guidelines proposed by Karoui et al. [35]. Generally, R_2_ values between 0.66 and 0.81 indicate a quantitative approximation to the reference value, while values between 0.82 and 0.90 suggest a good estimate, and values exceeding 0.91 indicate an excellent estimate. Moreover, RPD values below 1.9 are considered inadequate; values between 2.0 and 2.4 are considered poor and suitable only for preliminary screening; values between 2.5 and 2.9 may be used for screening purposes; and values exceeding 3 are deemed suitable for quality control and can substitute wet chemistry analysis [36]. Furthermore, for models achieving an RPD ≥ 2.0, systematic bias was assessed through paired *t*-tests (Table S2) and Bland–Altman analysis (Figure S1). These evaluations confirmed that the mean differences between predicted and reference values did not significantly differ from zero, indicating the absence of systematic over- or under-estimation across the investigated concentration ranges.

## 3. Results and Discussion

### 3.1. Study Limitations

This study assessed the feasibility of MIR and NIR to analyze PBB similarly to what is done in the dairy industry for milk and to provide guidance for future prediction model development. Therefore, as an exploratory study, some limitations have to be considered for the correct interpretation of the results.

The main limitation is the reduced number of samples. The sample size may be considered small for external validation of quantitative prediction models, but cross-validation is a valid approach to demonstrate the method’s feasibility and select which traits to prioritize in the future to increase the sample size, enabling an external evaluation. Other studies with similar sample sizes applied the cross-validation approach to demonstrate the capacity of MIR to properly discriminate among the main PBB categories (*n* = 40 samples, [20]). Moreover, a study conducted with 145 cheese samples demonstrated that both external validation and cross-validation approaches yielded similar results [34]. Nevertheless, the reported calibration statistics should be interpreted as preliminary until confirmed using an independent external validation dataset. Another limitation of the study was the wide array of formulations of the most common PBBs in the market that could impair global calibration models and transferability. To mitigate this limitation, different brands and different formulations of the same PBBs were selected for the present study.

### 3.2. Spectra and Canonical Discrimination Analysis

**Figure 1:**
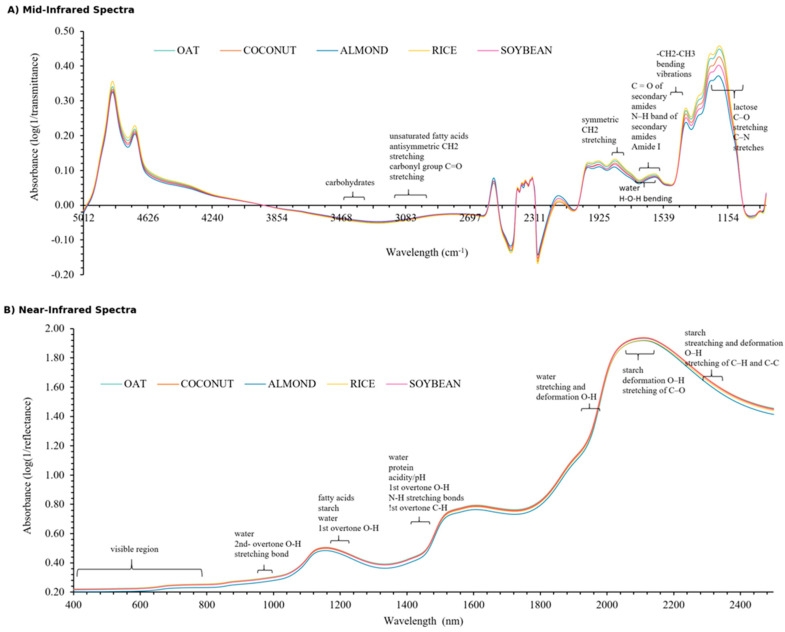
Average raw spectra of the different plant-based beverages as milk alternatives included in the present study using (**A**) mid-infrared and (**B**) near-infrared spectroscopy. The different categories are represented in different colors. Based on the literature, peaks or regions described in plant-based beverages have been included and more detailed information is provided in the main text.

Figure 1 displays the raw MIR (Figure 1A) and NIR (Figure 1B) spectra, revealing a similar trend across PBBs, which partially agreed with Brito et al. [20], where rice- and oat-based beverages showed a similar spectral profile, while soybean- and almond-based beverages showed distinct spectral profiles. Based on the scarce literature on the topic, the absorbance peaks and bands observed in the MIR region at 1250–1150 cm^−1^ and 1476–1151 cm^−1^ are related to axial C–O stretching absorption bands, which are linked to total sugar content [17]. Moreover, intervals 1680–1630 cm^−1^ (C=O of secondary amides), 1640–1550 cm^−1^ (N–H band of secondary amides), and 1350–1000 cm^−1^ (C–N stretches) are relevant for total protein content [16]. Moreover, a characteristic band of unsaturated fatty acids in soybean oil has been described at 3007 cm^−1^ [15]. Tulashie et al. [18] reported that 1637 cm^−1^ stretches in the MIR region are related to protein (Amide I), the band at 3350 cm^−1^ is linked to carbohydrates, and bands around 2853, 1743, and 2923 cm^−1^ could be antisymmetric and symmetric CH_2_ stretching and carbonyl group C=O stretching from fat. Sitorus and Bulan [19] also reported that in the MIR region, fatty acids are identified at 5741 nm (~1743 cm^−1^) and 6840 nm (~1463 cm^−1^) because these regions are associated with C=O from an ester of triacylglycerol and -CH_2_-CH_3_ bending vibrations, respectively. Absorption peaks of the protein components are identified around 6116 nm (~1636 cm^−1^), which refers to the functional group of C=O stretching vibrations and -NH bending vibrations [19]. Lactose absorption peaks are observed around 9285 nm (~1077 cm^−1^), corresponding to the –CO stretching vibrations of the functional group [19]. The fingerprint of water is observed at a wavelength of 6079 nm (~1645 cm^−1^) due to the absorbance peak of the H-O-H bending mode [19]. Moreover, in the NIR region, three peaks have been described in grated coconut samples for coconut-based beverages at 980 (water), 1210 (fatty acids), and 1450 (water/protein, acidity-pH) nm, corresponding to the second-order overtone vibration of the O-H stretching bond (980 nm) and the first-order overtones of O-H and N-H stretching bonds (1450 nm) [21]. In rice-based beverages, Silva et al. [23] described the spectrum and identified seven relevant NIR bands. Those refer to the vibrations of the stretching second overtone of C–H (8312 cm^−1^; ~1203 nm), stretching first overtone of O-H related to starch and water (6902 cm^−1^; ~1449 nm), stretching first overtone of C–H (5662 cm^−1^; ~1766 nm), stretching and deformation of O-H (5172 cm^−1^; ~1934 nm) probably related to water, deformation of O–H and stretching of C–O related to starch (4774 cm^−1^; ~2095 nm), stretching and deformation of C–H (4316 cm^−1^; ~2317 nm), and a band associated with the stretching of C–H and C–C related to starch.

**Figure 2:**
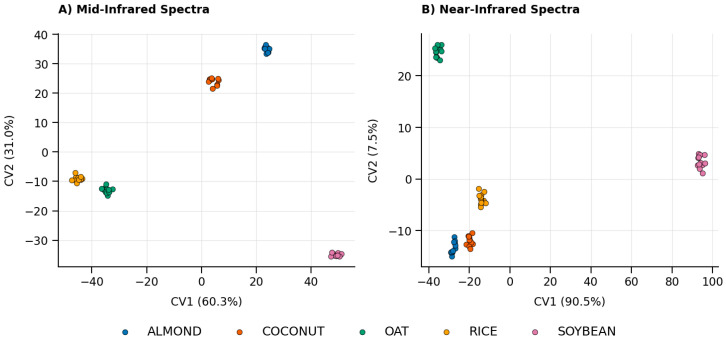
Canonical discrimination analysis score plots after wavelength selection of plant-based-beverage samples obtained from (**A**) mid-infrared (MIR) and (**B**) near-infrared (NIR) spectral information. Each point represents an individual sample, colored according to beverage category (almond, coconut, oat, rice, and soybean).

Canonical discrimination analysis performed on the MIR spectra using 30 wavelengths explained 91.3% of the total variance (Canonical function 1, 60.3%; Canonical function 2, 31.0%; Figure 2A). Of those, nine of the retained wavelengths were between 1476 and 1150 cm^−1^, which are linked to total sugar content [17]. The wavelength 1710 cm^−1^ was also retained, which has been associated with fat [18], as were wavelengths 1384 and 1432 cm^−1^, which were linked to lipid structures [37]. Wavelengths at 1080 and 1235 cm^−1^ have been associated with lactose and lipids [37]. Moreover, five wavelengths were kept between 4396 and 4488 cm^−1^, which fall within the region dominated by water (3008 and 5010 cm^−1^; [38]). Based on a PCA, Brito et al. [20] used spectral information between 1720.6 and 1371 cm^−1^ for classification purposes achieving a good separation of rice-, oat-, and soybean-based beverages, all of which suggest that water content, sugars, and fat are relevant for discriminating them. The score plot revealed that MIR spectra clearly discriminated among all PBB classes (Figure 2A).

The CDA performed on the NIR spectra using 35 wavelengths explained 98.0% of the total variance (Canonical function 1, 90.5%; Canonical function 2, 7.5% (Figure 2B)). Of those wavelengths, 13 were between 406 and 583 nm, corresponding to the visible spectrum and covering violet (380–450 nm), blue (450–485 nm), cyan (485-500 nm), green (500–565 nm), and yellow (565–590 nm) [39]. Wavelengths 969 nm and 1465 nm selected in the NIR region are closer to the water and protein bands (980 nm and 1450 nm; [21]), and the one selected at 1465 nm is closer to the stretching first overtone of C–H (1449 nm; [23]). Moreover, the ones at 2080 nm and 2398 are closer to the ones related to starch (2095 nm and 2317; [23]). This all suggests that the color of the PBB, water content, and starch are relevant for discriminating among them. The plot also clearly revealed the capability of NIR spectra to discriminate among the five PBB classes (Figure 2B). These findings are consistent with the recent study of Christodoulou et al. [40], who evaluated commercial almond-, oat-, rice-, and soy-based beverages using ATR-FTIR spectroscopy combined with chemometric approaches (PLS-DA and OPLS-DA). The authors reported clear separation among beverage categories and identified protein- and carbohydrate-associated spectral regions as the main contributors to discrimination, further supporting the use of infrared spectroscopy for the authentication and classification of PBBs.

Although in the present studies proper discrimination was achieved through wavelength selection and CDA in both technologies, MIR and NIR, de Paulo et al. [16] suggested a limitation of infrared spectroscopy when using PCA to discriminate among PBBs, due to different water proportions in each brand’s formula within the same variety. Moreover, Silva et al. [23] reported that the brand has a greater influence on the ability to distinguish rice-based beverage samples than their composition. Therefore, wavelength selection is recommended to build classificatory models.

The clear separation among PBB categories observed in the CDA score plots also suggests that future studies could benefit from developing category-specific calibration equations rather than a single global calibration across all PBBs. Similar strategies are routinely adopted in the dairy industry, where separate prediction equations have been developed for cow, goat, sheep, and buffalo milk to account for matrix-specific compositional and spectral differences.

### 3.3. Prediction Models for Gross Composition and Acidity Traits

Table 3 displays the performance of the infrared prediction models for gross composition and acidity traits. The number of outliers removed during model development ranged from 0 to 9. The removed samples were distributed across all PBB categories. Both instruments revealed a similar number of LFs retained for the prediction model: below 5 for MIR and below 6 for NIR, which is in agreement with previous studies in soybean-based beverages by Rech et al. [17], de Paulo et al. [16], and Williams [41], who used up to 10–12 LFs for developing proper calibration models. For both instruments, we also observe a decrease in the coefficient of determination from R^2^_C_ to R^2^_CV_ and an increase in the SE_CV_ [41]. Moreover, this shift was more pronounced when the models were developed using NIR spectra rather than MIR spectra.

Based on MIR spectra, excellent prediction models were obtained for protein content, with R^2^_CV_ > 0.99 and an RPD of 18.50, suggesting their applicability as replacements for the reference method. Good estimation models for quality control were achieved for fat, glucose, and ash content (RPD, 3.19–3.89). In contrast, the equations for fructose content and titratable acidity can only be considered suitable for screening purposes (RPD, 2.48–2.60). Poor prediction models were obtained for lactose. The limited predictive performance for lactose may be related to the fact that lactose is not an inherent component of a PBB. Lactose is a disaccharide uniquely synthesized by mammary epithelial cells and constitutes the principal carbohydrate of mammalian milk [42,43]. Consequently, lactose is not a natural constituent of a PBB, and its detection may be the result of dairy-derived ingredient addition, cross-contamination during manufacturing, or product adulteration/mislabeling. This heterogeneous and potentially sporadic presence of lactose among samples could have contributed to the poor calibration and prediction performance of the models. However, none of the products included in this study declared lactose as an ingredient on their labels (Table S1).

Poor prediction models were also obtained for pH. The limited predictive ability observed for this trait was likely attributable, at least in part, to the narrow range of reference values available in the dataset (mean = 6.60, SD = 0.31, CV = 5%; Table 1). The development of robust infrared calibration models requires sufficient variability in the reference data to establish reliable relationships between spectral information and the trait of interest, whereas traits characterized by a restricted range often yield reduced predictive performance [24,41]. Furthermore, pH is not a directly infrared-active property, as MIR and NIR spectroscopy measure molecular vibrations associated with chemical bonds rather than hydrogen ion activity [12,24]. Consequently, pH can only be estimated indirectly through spectral changes related to acid-base equilibria, water organization, and hydrogen-bonding interactions [12,24]. On the other hand, Suksangpanomrung et al. [21] achieved satisfactory pH predictions only when analyzing grated coconut materials characterized by considerably higher pH concentrations and wider compositional variability than those present in commercial coconut-based beverages, highlighting the importance of matrix characteristics and calibration range for successful pH prediction.

Therefore, MIR can be used to determine gross composition (i.e., protein, fat, glucose, and ash content), as is already done for milk [15]. In soybean-based beverages presenting a similar range of glucose content to ours, Rech et al. [17] achieved a slightly greater R^2^_C_ (0.98) than we did (0.95; Table 2) when applying MIR spectral region selection techniques. However, the slight improvement does not support the need for applying band selection, as those authors reported a greater LF (LF, 6–7) than we needed. A greater number of LF was also retained for soybean-based beverages by de Paulo et al. [16] for total protein (LF, 10) and total sugars (LF, 9) using the complete spectra. Moreover, the total protein prediction model accuracy in de Paulo et al. [16] was lower (R^2^_C_, 0.88) than the one reported in Table 3 with both techniques. Nevertheless, those authors developed a more accurate prediction model for total sugars (R^2^_C_, 0.99; [16]) than the one we reported for glucose content, particularly when compared with NIR (Table 3).

On the other hand, the NIR prediction models built were only adequate for quality control for protein content (RPD, 3.04) and screening purposes for ash content (RPD, 2.99). However, the developed prediction models can be applied for preliminary screening purposes for fat content, glucose content, and titratable acidity (RPD, 2.03–2.27). More accurate NIR models have been developed for grated coconut samples (both raw and defatted) from coconut-based beverages [21]. Those authors built excellent prediction models for protein content (RPD, 5.83), fat content (RPD, 7.72), and pH (RPD, 3.70). However, they did not build the models on the coconut-based beverage, but rather on the raw and defatted coconut samples, which had greater fat and protein content and range (Fat, 15–33%; Protein, 3–9%) than the PBBs. The concentration of a trait, as well as covering a more comprehensive range, is crucial to developing robust calibration models [24].

### 3.4. Prediction Models for Detailed Composition

To our knowledge, this is the first study to develop infrared-based prediction models for fatty acids, minerals, and amino acids content in PBBs. Therefore, the discussion will be based on other presentations of the main PBB ingredient, such as flakes, cakes, or flour, and in milk and dairy products, as PBBs are proposed as milk substitutes. Moreover, as previously mentioned, due to the very low fat content (Table 1), predictions for fatty acids are not reported as we were below the limit of detection for infrared spectroscopy (0.1%) for almost all of them.

Table 4 displays the performance of the infrared prediction models for mineral content. The number of outliers removed to build the models ranged from 2 to 6. The removed samples were distributed across all categories. In general, the LF was greater for minerals than for the previous traits, which is in agreement with earlier studies on milk [43] and dairy products [24]. A similar range of LFs was retained with both techniques, NIR and MIR, reaching up to 7. Prediction models were better for MIR than for NIR. For MIR, P (R^2^_CV_, 0.95; RPD, 4.54) and K (R^2^_CV_, 0.97; RPD, 5.69) revealed good estimation models for quality control, while it was not possible to build adequate calibration models to predict I, Ca, Mg, and Na. Moreover, the only minerals that NIR was able to predict to some degree were P and K. For P (R^2^_CV_, 0.86; RPD, 2.72), the prediction model can be considered suitable for screening purposes, whereas the one for K (R^2^_CV_, 0.78; RPD, 2.16) might be regarded as only for preliminary screening. In milk, reported prediction models have shown R^2^ values ranging from 0.25 to 0.87 [44]. However, some authors have reported slightly better results for Ca, Na, and P with RPD, ranging from 2.07 to 2.41 [30]. On the other hand, more accurate prediction models adequate for quality control have been developed using NIR in transmittance mode for some minerals such as Ca, P, S, Mg, Zn, and Cu (RPD, 3.02–3.73) because they are bonded to the casein micelle [24].

Table 5 displays the performance of the infrared prediction models for amino acid content. The number of outliers removed to build the models ranged from 0 to 9. Those samples eliminated were across all categories. Only for glutamic acid was the MIR model developed using 45 samples. However, to be able to compare both technologies, this trait was kept. Both devices showed a similar LF range for building prediction models for amino acids, ranging from 1 to 7. As observed for the other traits, prediction models were more accurate for MIR than for NIR. The only amino acid that showed poor prediction capability for MIR was phenylalanine (R^2^_CV_, 0.46; RPD, 1.38). All the other amino acids had excellent prediction models (R^2^_CV_, 0.92 to 0.99) that could be applied for quality control or any other application. Moreover, most achieved R^2^_CV_ values greater than 0.98 and RPD values ranging from 5 to 15. On the other hand, less accurate prediction models were built with NIR spectra. Phenylalanine (R^2^_CV_, 0.45; RPD, 1.36) and histidine (R^2^_CV_, 0.73; RPD, 1.96) revealed poor predictability with NIR. Adequate prediction models for screening purposes were achieved for arginine, serine, glutamic, and methionine (RPD, 2.86–2.98). All the other amino acid prediction models were good enough to be applied for quality control (RPD, 3.03–3.59). However, the accuracy of the models was lower than that of the ones developed with MIR.

The strong predictive performance likely reflects the close relationship between individual amino acid concentrations and total protein content across the studied beverages. Since amino acids are structural constituents of proteins, a substantial proportion of the predictive ability may arise from protein-associated spectral information rather than from unique amino-acid-specific absorption bands. This interpretation is consistent with studies showing that amino-acid predictions are strongly linked to the protein matrix and that protein infrared spectra are dominated by amide-related absorptions, whereas signals from individual amino-acid side chains are comparatively smaller and often overlapping [45,46]. The good predictability of amino acids in PBBs using infrared spectroscopy contrasts with previous studies on milk. Poor prediction models have been reported by McDermott et al. [47] for free amino acids, with RPD values ranging from 1.07 (serine) to 1.38 (glycine).

Phenylalanine was the only amino acid that could not be accurately predicted by either MIR or NIR spectroscopy. A plausible explanation is that infrared-based prediction of individual amino acids is largely indirect and relies on correlations with protein spectral features rather than on unique amino-acid-specific absorption bands, because protein infrared spectra are dominated by the amide I and amide II regions, and the contributions of individual amino-acid side chains are comparatively small and often overlap with broader protein absorptions [45]. Moreover, phenylalanine is an aromatic amino acid whose spectral contributions may overlap with those of other aromatic residues and protein-related signals, limiting the amount of independent information available for calibration. In addition, the relationship between phenylalanine concentration and the overall protein matrix may have been less consistent across the different plant sources included in this study, thereby reducing model robustness. Similar limitations have been reported when interpreting protein infrared spectra, where amino-acid side-chain contributions are often masked by dominant amide absorptions and overlapping vibrational bands [45].

## 4. Conclusions

In conclusion, this study suggests that both MIR and NIR spectroscopy can adequately discriminate among the five most consumed plant-based beverages: oats, almonds, soybeans, rice, and coconuts. Regarding the quantitative models, although both MIR and NIR demonstrated the feasibility of predicting the studied traits, MIR outperformed NIR spectroscopy in terms of the accuracy of the models. Our results suggest that MIR could also be implemented to accurately predict total ashes, P and K, protein, and amino acid profile. In fact, the best prediction models were achieved for total protein and all amino acids except phenylalanine. The main limitation of the present study was the small number of samples available for quantitative analysis, given the high number of analyses performed. However, the excellent performance of some of the predicted models (gross composition, detailed amino acid profiles, and some minerals) may support the integration of MIR into routine quality-control workflows for plant-based beverages as milk alternatives, leveraging existing dairy-industry infrastructure and enhancing analytical capabilities for emerging non-dairy products. Such implementation is consistent with the increasing adoption of infrared spectroscopy as a Process Analytical Technology for real-time quality monitoring in food and dairy processing environments. Future investigations should confirm these findings for the most promising traits by increasing the number of samples to enable external validation, and explore additional chemometric strategies to improve the prediction of low-abundance constituents, such as certain fatty acids. Future studies should also evaluate whether category-specific calibrations for individual plant-based beverage types can improve prediction performance compared with the global calibration approach adopted in the present work.

## Figures and Tables

**Table 1:** Descriptive statistics ^1^ for gross chemical composition, sugar profile, minerals and amino acids of plant-based beverages (PBBs) as milk alternatives included in the present study (*n* = 57).

Constituent	Mean	SD	Minimum	Maximum	CV, %
Gross composition, %					
Protein	1.12	1.25	0.06	3.90	112
Fat	1.28	0.99	0.11	3.85	77
Lactose	1.18	1.32	0	5.05	112
Glucose	1.72	1.94	0.02	6.78	113
Fructose	0.68	1.21	0.02	5.66	178
Ash	0.21	0.14	0.05	0.62	67
Acidity					
pH	6.60	0.31	5.97	7.27	5
Titratable acidity, SH°	0.85	0.83	0.18	3.78	98
Minerals					
Iodine, µg/kg PBB	7.42	10.99	0.19	65.00	148
Calcium, mg/kg PBB	188.39	79.68	44.00	389.00	42
Phosphorus, mg/kg PBB	192.25	176.16	29.00	723.00	92
Magnesium, mg/kg PBB	2157.67	3335.95	11.00	9884.00	155
Potassium, mg/kg PBB	500.56	494.13	108	2052.00	99
Sodium, mg/kg PBB	312.28	128.27	70.00	584.00	41
Sulphur, mg/kg PBB	125.62	95.87	10.00	264.00	76
Amino acids, mg/100 g PBB					
Histidine	27.77	31.35	1.54	116.28	113
Arginine	84.47	89.53	0.96	325.25	106
Serine	51.34	58.42	0.59	193.23	114
Glycine	47.71	46.18	1.69	148.38	97
Aspartic	130.42	154.54	4.49	487.07	118
Glutamic	278.56	273.69	1.32	867.02	98
Threonine	35.76	44.23	0.94	141.24	124
Alanine	45.67	48.86	1.41	155.82	107
Proline	48.23	57.57	0.34	180.05	119
Lysine	68.10	100.95	0.20	322.3	148
Methionine	7.83	9.53	0.00	41.04	122
Tyrosine	31.42	39.86	0.01	141.75	127
Valine	39.88	44.22	0.02	143.57	111
Cystine	10.79	9.66	1.31	33.69	90
Isoleucine	37.42	48.05	0.60	145.81	128
Leucine	73.23	82.58	1.63	270.76	113
Phenylalanine	32.20	45.22	0.37	199.92	140
Tryptophan	13.75	16.82	0.18	56.61	122

^1^ SD = standard deviation; CV = coefficient of variation.

**Table 2:** Descriptive statistics ^1^ for the fatty acids profile and content of plant-based beverages (PBBs) as milk alternatives included in the present study (*n* = 57).

	Fatty Acids, %					Fatty Acids, g/100 g PBB				
Constituent ^2^	Mean	SD	Min	Max	CV, %	Mean	SD	Min	Max	CV, %
C8:0	1.37	2.91	0	8.81	212	0.020	0.040	0	0.11	200
C10:0	1.03	2.21	0	6.3	215	0.010	0.030	0	0.08	300
C12:0	7.88	17.17	0	49.07	218	0.100	0.220	0	0.63	220
C14:0	3.19	6.49	0	18.92	203	0.040	0.080	0	0.25	200
C16:0	11.27	4.80	5.68	29.41	43	0.150	0.060	0.073	0.38	40
C16:1n9	0.09	0.16	0	0.68	178	0.001	0.002	0	0.01	200
C16:1n7	0.18	0.20	0	0.84	111	0.002	0.003	0	0.01	150
C17:0	0.06	0.05	0	0.19	83	0.001	0.001	0	0.002	100
C18:0	3.21	1.66	1.16	8.60	52	0.040	0.020	0.015	0.11	50
C18:1n9	39.77	22.82	4.11	84.52	57	0.510	0.300	0.053	1.09	59
C18:1n7	0.95	0.50	0.05	2.83	53	0.010	0.010	0.001	0.04	100
C18:2n6	27.31	19.15	0	56.96	70	0.350	0.250	0	0.74	71
C18:3n3	2.06	3.34	0	13.76	162	0.030	0.043	0	0.18	143
C19:1	0.10	0.16	0	0.65	160	0.001	0.002	0	0.01	200
C20:0	0.31	0.25	0	1.57	81	0.004	0.003	0	0.02	75
C20:1n9+C20:1n7	0.29	0.36	0	1.39	124	0.001	0.002	0	0.01	200
C22:0	0.34	0.38	0	1.91	112	0.004	0.010	0	0.02	250
C24:0	0.17	0.22	0	0.84	129	0.004	0.010	0	0.03	250
SFA, %	31.25	27.82	0	88.82	89	0.404	0.360	0	1.15	89
MUFA, %	38.78	23.29	0	86.94	60	0.500	0.300	0	1.12	60
PUFA, %	24.41	21.19	0	66.02	87	0.320	0.270	0	0.85	84
n-3	1.88	3.04	0	13.76	162	0.020	0.040	0	0.18	200
n-6	22.59	19.46	0	56.96	86	0.290	0.250	0	0.74	86

^1^ SD = standard deviation; Min = Minimum; Max = Maximum; CV = Coefficient of Variation. ^2^ SFA = saturated fatty acids; MUFA = monounsaturated fatty acids; PUFA = polyunsaturated fatty acids.

**Table 3:** Best prediction models for mid- (FT-MIR) and near-infrared (NIR) spectroscopy statistics ^1^, based on 5-fold cross-validation modified partial least-squares regression model, for gross chemical composition and acidity traits of plant-based beverages as milk alternatives. Best predicted traits (residual predictive deviation > 2.00) for at least preliminary screening application are in bold.

Constituent	*n*	Scatter Correction ^2^	Mathematical Treatment ^3^	LF	R^2^_C_	SE_C_	R^2^_CV_	SE_CV_	RPD
FT-MIR, 5000–900 cm^−1^									
**Protein, %**	**49**	**None**	**1,4,4,1**	**2**	**0.998**	**0.05**	**0.997**	**0.07**	**18.50**
**Fat, %**	**48**	**None**	**0,0,1,1**	**1**	**0.93**	**0.20**	**0.90**	**0.24**	**3.19**
Lactose, %	56	None	2,5,5,1	4	0.74	0.62	0.72	0.63	1.90
**Glucose, %**	**55**	**Detrend**	**2,10,10,1**	**4**	**0.95**	**0.37**	**0.92**	**0.48**	**3.51**
**Fructose, %**	**53**	**Detrend**	**0,0,1,1**	**2**	**0.91**	**0.14**	**0.83**	**0.19**	**2.48**
**Ash, %**	**54**	**None**	**1,4,4,1**	**5**	**0.97**	**0.03**	**0.93**	**0.04**	**3.89**
pH	52	Detrend	2,5,5,1	4	0.58	0.19	0.27	0.25	1.18
**Titratable acidity, SH°**	**54**	**Detrend**	**1,4,4,1**	**4**	**0.90**	**0.18**	**0.85**	**0.22**	**2.60**
NIR, 400 nm–2500 nm									
**Protein, %**	**55**	**Detrend**	**2,10,10,1**	**3**	**0.95**	**0.28**	**0.89**	**0.40**	**3.04**
**Fat, %**	**50**	**Detrend**	**1,4,4,1**	**1**	**0.93**	**0.26**	**0.80**	**0.42**	**2.27**
Lactose, %	56	MSC	2,5,5,1	3	0.48	0.88	0.39	0.94	1.30
**Glucose, %**	**57**	**None**	**0,0,1,1**	**2**	**0.83**	**0.79**	**0.77**	**0.91**	**2.11**
Fructose, %	56	SNV	1,8,8,1	2	0.90	0.30	0.63	0.57	1.66
**Ash, %**	**53**	**None**	**1,8,8,1**	**2**	**0.93**	**0.03**	**0.88**	**0.04**	**2.90**
pH	50	Detrend	1,8,8,1	6	0.39	0.24	0.20	0.27	1.13
**Titratable acidity, SH°**	**54**	**Detrend**	**2,10,10,1**	**3**	**0.95**	**0.13**	**0.75**	**0.28**	**2.03**

^1^ *n* = number of samples; LF = latent factor; R^2^_C_ = coefficient of determination of calibration; SE_C_ = standard error of calibration; R^2^_CV_ = coefficient of determination of cross-validation; SE_CV_ = standard error of cross-validation; RPD = residual predictive deviation. ^2^ SNV = standard normal variate; SNV + D = standard normal variate and detrending; MSC = multiplicative scatter correction. ^3^ The first digit indicates the derivative treatment, the second digit denotes the gap over which the derivative is computed, the third digit refers to the number of data points in the first smoothing, and the fourth digit indicates the number of data points in the second smoothing.

**Table 4:** Best prediction models for mid- (FT-MIR) and near-infrared (NIR) spectroscopy fit statistics ^1^, based on 5-fold cross-validation modified partial least-squares regression model, for mineral content of plant-based beverages as milk alternatives. Best predicted traits (residual predictive deviation > 2.00) for at least preliminary screening application are in bold.

Constituent	*n*	Scatter Correction ^2^	Mathematical Treatment ^3^	LF	R^2^_C_	SE_C_	R^2^_CV_	SE_CV_	RPD
FT-MIR, 5000–900 cm^−1^									
Iodine, µg/kg	51	None	1,8,8,1	5	0.60	2.66	0.12	3.89	1.08
Calcium, mg/kg	53	Detrend	2,5,5,1	5	0.85	30.16	0.58	49.46	1.55
**Phosphorus**, mg/kg	**52**	**Detrend**	**0,0,1,1**	**7**	**0.96**	**32.41**	**0.95**	**33.79**	**4.54**
Magnesium, mg/kg	51	MSC	2,10,10,1	2	0.60	2140.33	0.46	2450.32	1.38
**Potassium**, mg/kg	**52**	**None**	**1,4,4,1**	**5**	**0.99**	**48.64**	**0.97**	**74.63**	**5.69**
Sodium, mg/kg	55	Detrend	1,4,4,1	4	0.71	68.56	0.37	99.44	1.27
NIR, 400 nm–2500 nm									
Iodine, µg/kg	53	None	2,10,10,1	3	0.55	2.97	0.27	3.74	1.18
Calcium, mg/kg	51	MSC	1,4,4,1	7	0.87	29.71	0.46	58.94	1.37
**Phosphorus**, mg/kg	**53**	**Detrend**	**2,10,10,1**	**3**	**0.93**	**38.17**	**0.86**	**53.92**	**2.72**
Magnesium, mg/kg	53	Detrend	2,5,5,1	3	0.67	1927.84	0.34	2676.71	1.25
**Potassium**, mg/kg	**52**	**SNV + D**	**2,5,5,1**	**3**	**0.93**	**108.93**	**0.78**	**186.12**	**2.16**
Sodium, mg/kg	55	Detrend	2,10,10,1	3	0.55	87.03	0.02	127.35	1.02

^1^ *n* = number of samples; LF = latent factor; R^2^_C_ = coefficient of determination of calibration; SE_C_ = standard error of calibration; R^2^_CV_ = coefficient of determination of cross-validation; SE_CV_ = standard error of cross-validation; RPD = residual predictive deviation. ^2^ SNV = standard normal variate; SNV + D = standard normal variate and detrending; MSC = multiplicative scatter correction. ^3^ The first digit indicates the derivative treatment, the second digit denotes the gap over which the derivative is computed, the third digit refers to the number of data points in the first smoothing, and the fourth digit indicates the number of data points in the second smoothing.

**Table 5:** Mid- (FT-MIR) and near-infrared (NIR) spectroscopy best prediction models fit statistics ^1^, based on 5-fold cross-validation-modified partial least-squares regression model, for amino acid composition of plant-based beverages (mg/100 g PBB) as milk alternatives. Best predicted traits (residual predictive deviation > 2.00) for at least preliminary screening application are in bold.

Constituent	***n***	Scatter Correction ^2^	Mathematical Treatment ^3^	LF	R^2^_C_	SE_C_	R^2^_CV_	SE_CV_	RPD
FT-MIR, 5000–900 cm^−1^									
**Histidine**	**51**	**None**	**2,5,5,1**	**4**	**0.98**	**4.50**	**0.98**	**4.96**	**6.47**
**Arginine**	**51**	**Detrend**	**2,10,10,1**	**4**	**0.99**	**5.10**	**0.99**	**8.10**	**10.26**
**Serine**	**48**	**None**	**0,0,1, 1**	**1**	**0.99**	**3.89**	**0.99**	**4.67**	**13.32**
**Glycine**	**48**	**None**	**0,0,1,1**	**1**	**0.99**	**4.92**	**0.99**	**5.83**	**8.28**
**Aspartic**	**51**	**Detrend**	**0,0,1,1**	**2**	**0.99**	**8.19**	**0.99**	**11.22**	**14.10**
**Glutamic**	**45**	**None**	**0,0,1,1**	**1**	**0.99**	**21.29**	**0.99**	**27.33**	**10.82**
**Threonine**	**55**	**SNV**	**2,10,10,1**	**7**	**0.99**	**5.20**	**0.96**	**8.48**	**5.29**
**Alanine**	**49**	**None**	**2,10,10,1**	**5**	**0.99**	**3.35**	**0.99**	**4.36**	**11.61**
**Proline**	**54**	**Detrend**	**1,8,8,1**	**6**	**0.99**	**3.29**	**0.99**	**4.04**	**14.62**
**Lysine**	**50**	**Detrend**	**1,4,4,1**	**4**	**0.99**	**8.31**	**0.99**	**11.17**	**8.99**
**Methionine**	**49**	**Detrend**	**2,10,10,1**	**5**	**0.97**	**1.05**	**0.92**	**1.84**	**3.53**
**Tyrosine**	**52**	**None**	**1,4,4,1**	**5**	**0.99**	**2.86**	**0.99**	**3.52**	**10.87**
**Valine**	**52**	**Detrend**	**1,8,8,1**	**6**	**0.99**	**4.04**	**0.98**	**5.50**	**7.94**
**Cystine**	**57**	**Detrend**	**2,10,10,1**	**5**	**0.97**	**1.68**	**0.92**	**2.74**	**3.53**
**Isoleucine**	**50**	**Detrend**	**0,0,1,1**	**7**	**0.99**	**4.46**	**0.99**	**5.68**	**8.63**
**Leucine**	**51**	**None**	**1,8,8,1**	**5**	**0.99**	**4.02**	**0.99**	**5.67**	**14.05**
Phenylalanine	54	None	1,4,4,1	5	0.54	31.01	0.46	33.19	1.38
**Tryptophan**	**49**	**Detrend**	**1,4,4,1**	**4**	**0.99**	**1.05**	**0.99**	**1.51**	**11.15**
NIR, 400 nm–2500 nm									
Histidine	53	Detrend	2,10,10,1	3	0.85	11.81	0.73	15.46	1.96
**Arginine**	**55**	**Detrend**	**1,8,8,1**	**1**	**0.95**	**20.43**	**0.88**	**31.30**	**2.90**
**Serine**	**54**	**None**	**2,10,10,1**	**3**	**0.95**	**12.09**	**0.89**	**19.03**	**2.98**
**Glycine**	**55**	**None**	**2,10,10,1**	**1**	**0.96**	**9.87**	**0.89**	**15.13**	**3.11**
**Aspartic**	**56**	**None**	**2,10,10,1**	**5**	**0.97**	**28.57**	**0.91**	**47.17**	**3.30**
**Glutamic**	**54**	**SNV + D**	**1,8,8,1**	**7**	**0.96**	**56.18**	**0.88**	**98.38**	**2.86**
**Threonine**	**54**	**SNV + D**	**2,10,10,1**	**5**	**0.99**	**4.70**	**0.92**	**11.84**	**3.50**
**Alanine**	**54**	**Detrend**	**2,10,10,1**	**5**	**0.95**	**10.42**	**0.89**	**15.78**	**3.04**
**Proline**	**54**	**Detrend**	**2,10,10,1**	**5**	**0.98**	**8.90**	**0.91**	**16.58**	**3.39**
**Lysine**	**55**	**MSC**	**2,5,5,1**	**4**	**0.99**	**11.35**	**0.89**	**32.53**	**3.03**
**Methionine**	**51**	**None**	**2,10,10,1**	**5**	**0.99**	**0.78**	**0.88**	**2.31**	**2.90**
**Tyrosine**	**54**	**MSC**	**2,10,10,1**	**5**	**0.99**	**4.30**	**0.91**	**10.87**	**3.29**
**Valine**	**55**	**MSC**	**2,10,10,1**	**5**	**0.99**	**3.88**	**0.91**	**12.91**	**3.40**
**Cystine**	**51**	**Detrend**	**2,10,10,1**	**5**	**0.99**	**0.81**	**0.92**	**2.42**	**3.61**
**Isoleucine**	**53**	**None**	**2,10,10,1**	**5**	**0.97**	**7.67**	**0.90**	**14.48**	**3.16**
**Leucine**	**54**	**Detrend**	**2,10,10,1**	**5**	**0.99**	**9.84**	**0.92**	**22.47**	**3.59**
Phenylalanine	50	SNV	1,4,4,1	2	0.52	28.37	0.45	30.08	1.36
**Tryptophan**	**52**	**None**	**2,5,5,1**	**3**	**0.95**	**3.58**	**0.91**	**4.73**	**3.38**

^1^ *n* = number of samples; LF = latent factor; R2C = coefficient of determination of calibration; SEC = standard error of calibration; R2CV = coefficient of determination of cross-validation; SECV = standard error of cross-validation; RPD = residual predictive deviation. ^2^ SNV = standard normal variate; SNV + D = standard normal variate and detrending; MSC = multiplicative scatter correction. ^3^ The first digit indicates the derivative treatment, the second digit denotes the gap over which the derivative is computed, the third digit refers to the number of data points in the first smoothing, and the fourth digit indicates the number of data points in the second smoothing.

## Data Availability

The data presented in this study are available on request from the corresponding author due to privacy restrictions.

## Supplementary Materials

The following supporting information can be downloaded at: https://www.mdpi.com/article/10.3390/foods15152588/s1. Figure S1: Bland–Altman plot of near-infrared spectroscopy (NIR) and mid-infrared spectroscopy (MIR). *Y*-axis represents the difference between the paired measurements (e.g., reference value minus infrared predicted value). *X*-axis represents the average of the two paired measurements. The solid red line indicates the mean bias between methods. The dashed red lines represent the 95% limits of agreement (mean bias ± 1.96 SD), and the dotted green lines indicate the corresponding confidence intervals for the limits of agreement. The solid blue line represents the line of no difference (zero bias), indicating perfect agreement between the methods; Table S1: Ingredients list as reported in the label of the products grouped by categories (N = 57); Table S2: Significance of the T-test of the best predicted models (RPD > 2) for both infrared technologies.
