# Supplementary material for: Assessing the Feasibility of Mid- and Near-Infrared Spectroscopy for Classifying Plant-Based Beverages and Predicting Nutrient Content
Source: Foods. 2026 Jul 23;15(15):2588. doi: 10.3390/foods15152588 (PMC13464381; doi:10.3390/foods15152588)

Table S1. Ingredients list as reported in the label of the products grouped by categories (N = 57).

Category	n	List of Ingredients
Oat	12	Water; organic oats (10–16%); sometimes organic sunflower oil; sea salt; occasionally agave fibre
Almond	12	Water; almonds (3–11%); sugar or organic cane sugar; sea salt; emulsifiers (sunflower lecithin, E473/sucrose esters); stabilisers (xanthan gum); natural flavourings
Soybean	12	Water; organic soybeans or dehulled soybeans (5.6–13.5%); sea salt; occasionally sugar, vegetable fibre and xanthan gum
Rice	11	Water; rice (14–17%); rice oil or sunflower oil; sea salt
Coconut	10	Water; coconut water, coconut milk or coconut paste; sea salt; starches (rice or tapioca); stabilisers (gellan gum, guar gum, xanthan gum, carrageenan/E407, E412, E473); natural coconut flavouring

Table S2. Significance of the T-test of the best predicted models (RPD > 2) for both infrared technologies.

Constituent	MIR	NIR
Gross composition, %		
Protein	0.999	0.999
Fat	0.994	0.604
Glucose	1.000	0.974
Fructose	1.000	
Ash	1.000	0.998
Acidity		
Titratable acidity, SH°	1.000	1.000
Minerals		
Phosphorus, mg/kg PBB	1.000	1.000
Potassium, mg/kg PBB	1.000	0.094
Amino acids, mg/100g PBB		
Histidine	1.000	
Arginine	1.000	1.000
Serine	0.881	1.000
Glycine	1.000	1.000
Aspartic	1.000	1.000
Glutamic	1.000	1.000
Threonine	1.000	1.000
Alanine	1.000	1.000
Proline	1.000	1.000
Lysine	1.000	1.000
Methionine	1.000	1.000
Tyrosine	1.000	1.000
Valine	1.000	1.000
Cystine	1.000	1.000
Isoleucine	1.000	1.000
Leucine	1.000	1.000
Tryptophan	1.000	0.467

MIR = mid-infrared spectroscopy; NIR = near-infrared spectrocopy; RPD = residual predictive deviation

Figure S1. Bland–Altman plot of near-infrared spectroscopy (NIR) and mid-infrared spectrocopy (MIR). Y-axis represents the difference between the paired measurements (e.g., reference value minus infrared predicted value). X-axis represents the average of the two paired measurements. The solid red line indicates the mean bias between methods. The dashed red lines represent the 95% limits of agreement (mean bias ± 1.96 SD), and the dotted green lines indicate the corresponding confidence intervals for the limits of agreement. The solid blue line represents the line of no difference (zero bias), indicating perfect agreement between the methods.

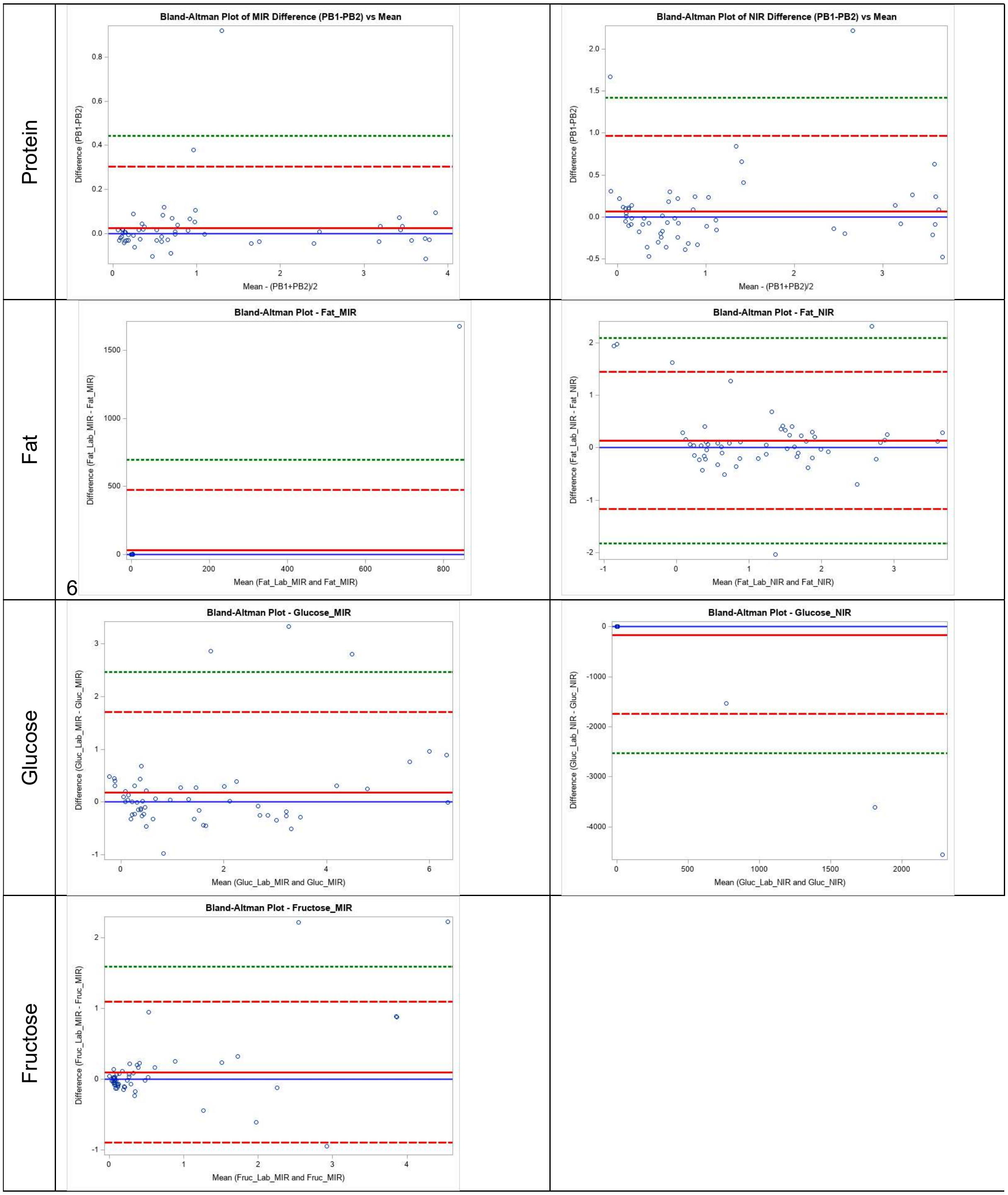

Ash

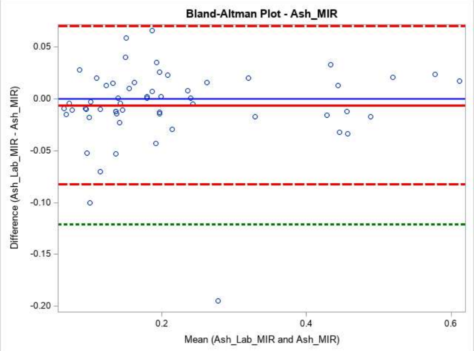

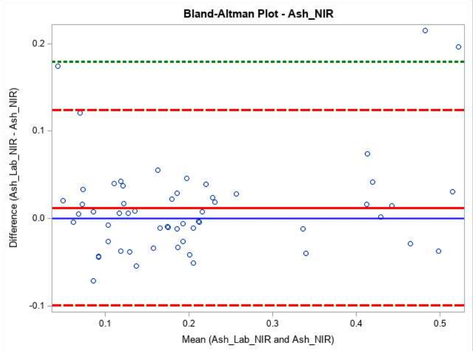

Titratable acidity, SH°

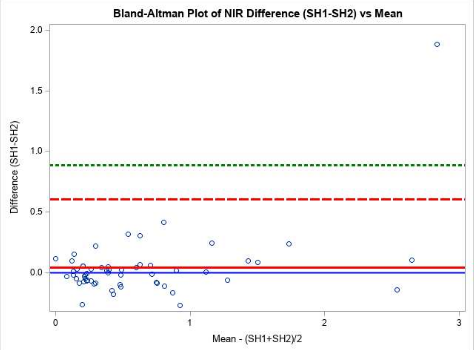

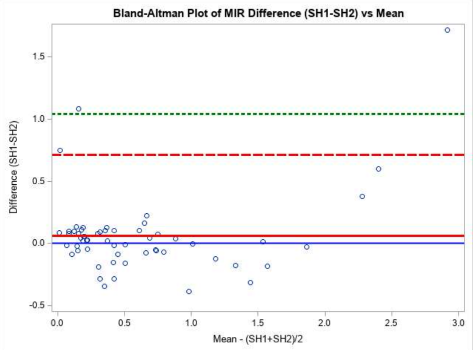

Phosphorus

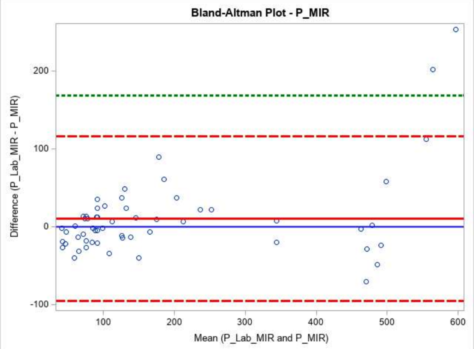

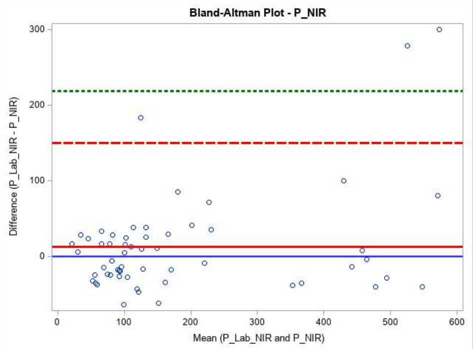

Potassium

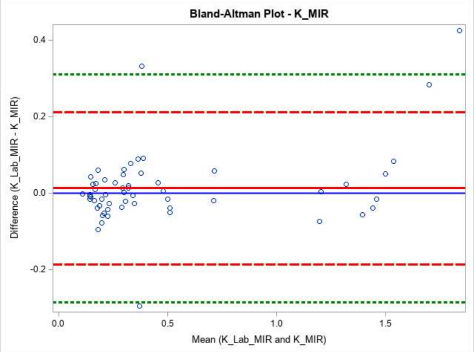

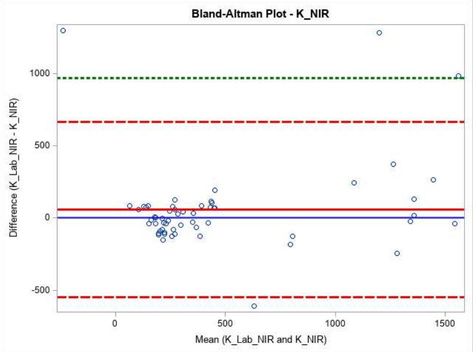

Histidine

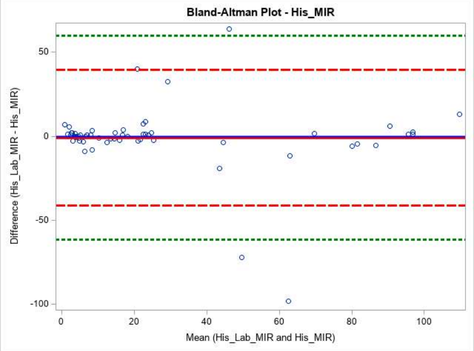

Argininge

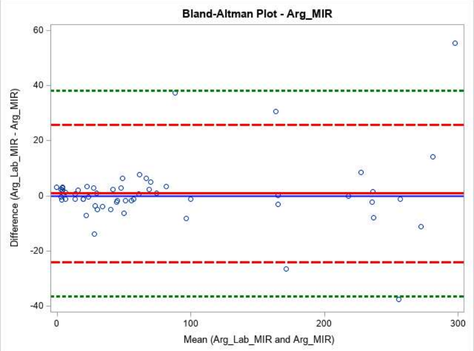

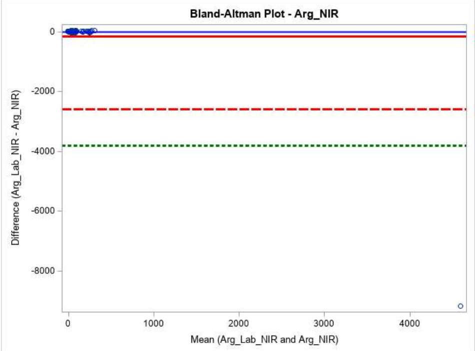

Serine

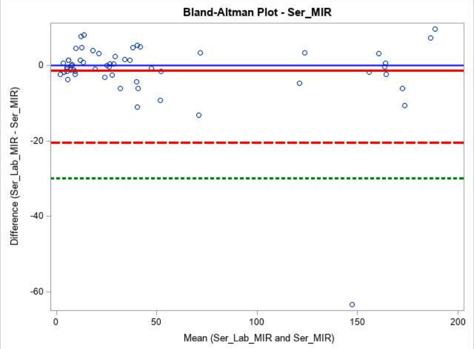

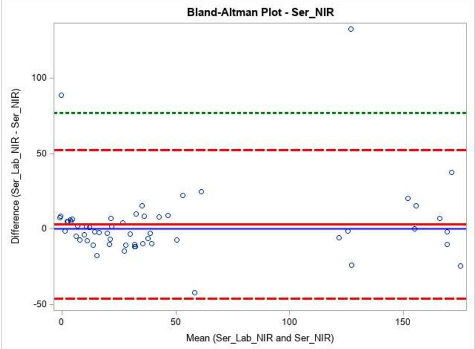

Glycine

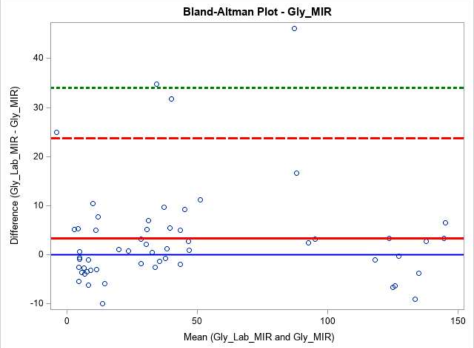

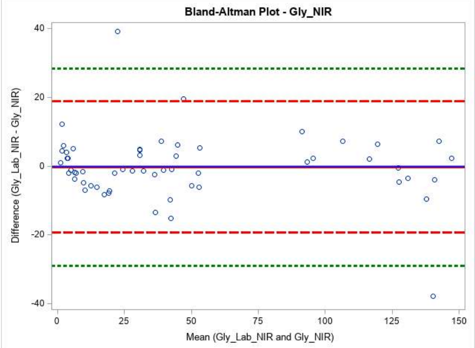

Aspartic

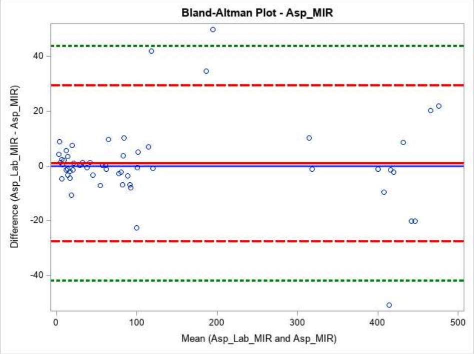

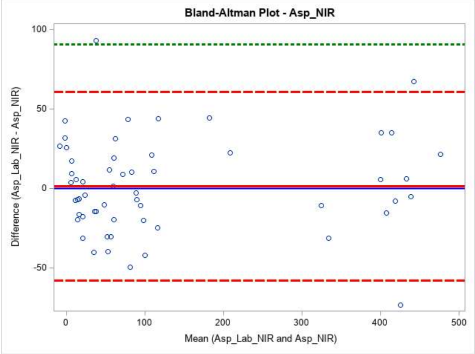

Glutamic	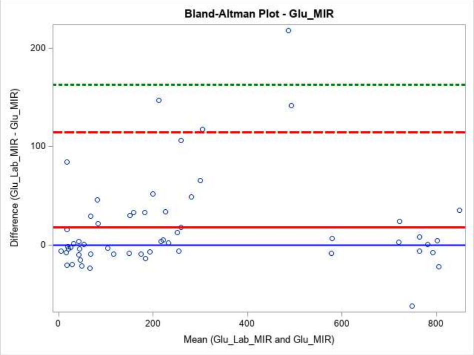	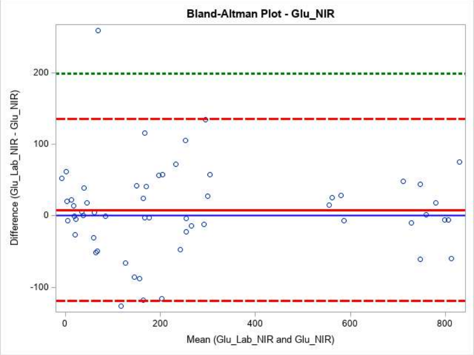
Threonine	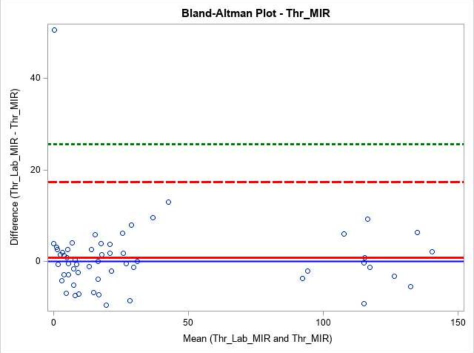	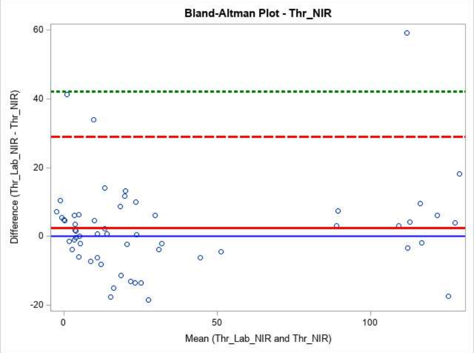
Alanine	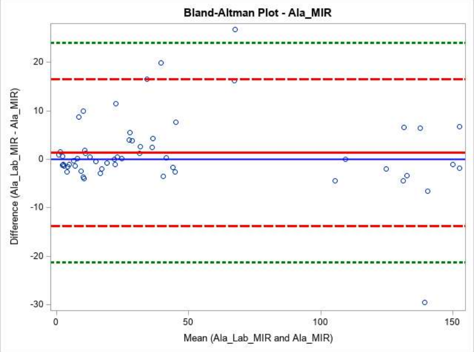	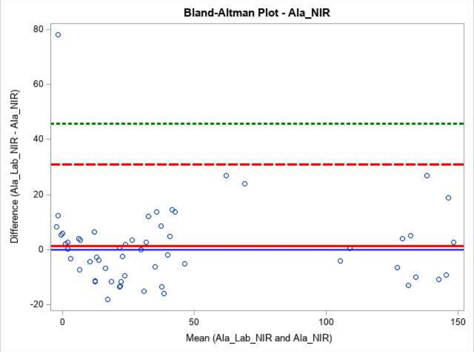
Proline	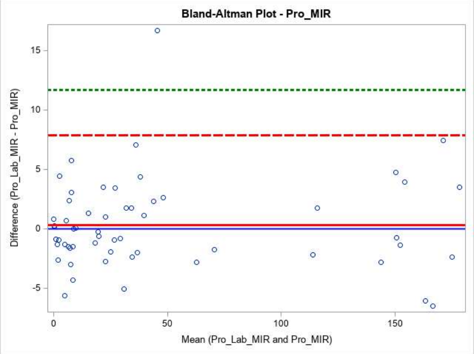	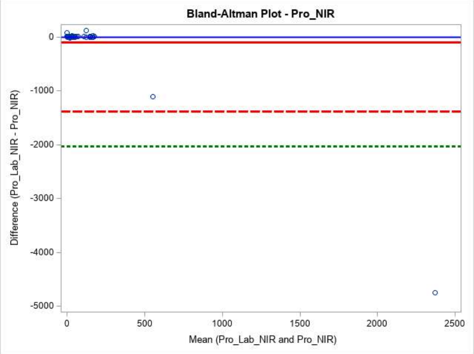
Lysine	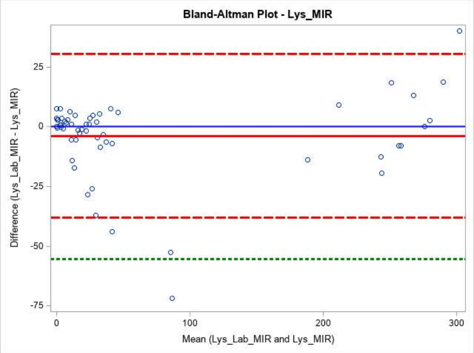	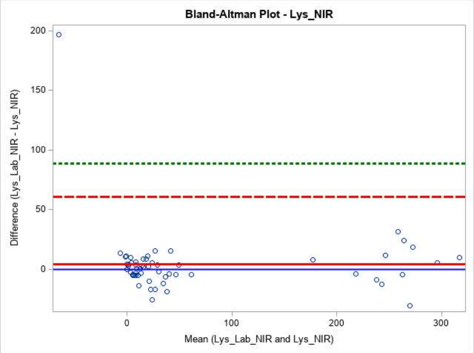

Methinine

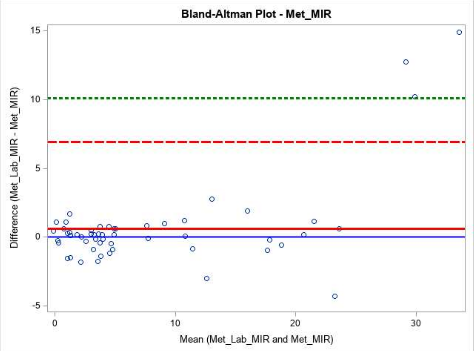

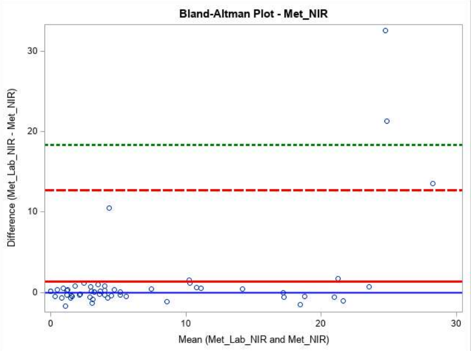

Tyrosine

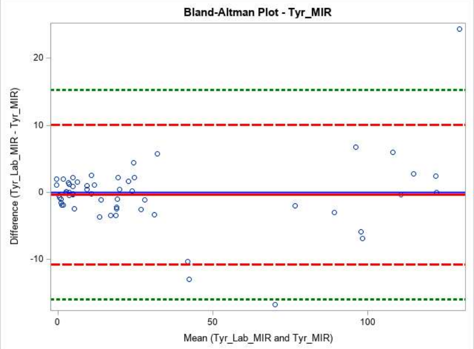

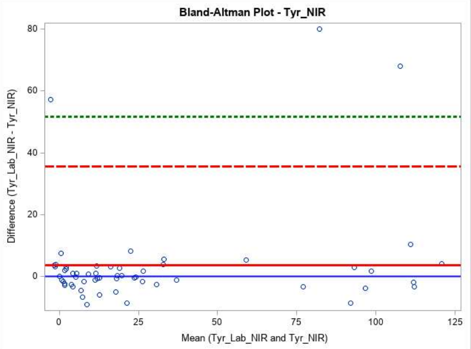

Valine

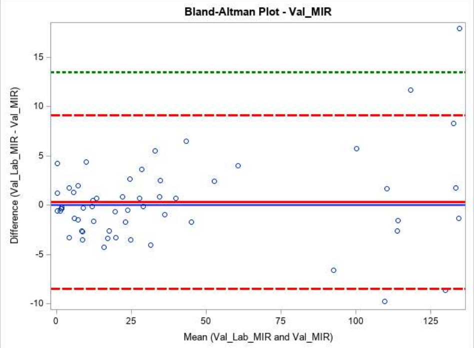

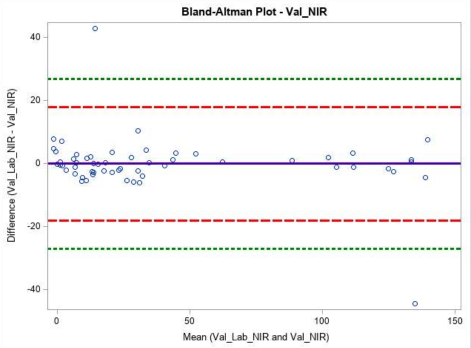

Cystine

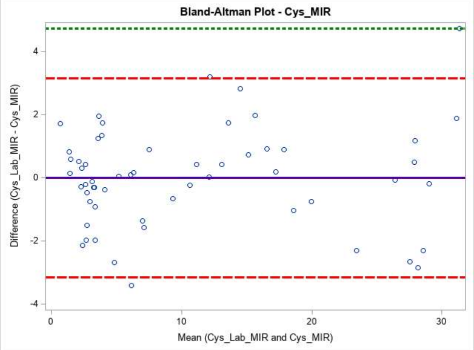

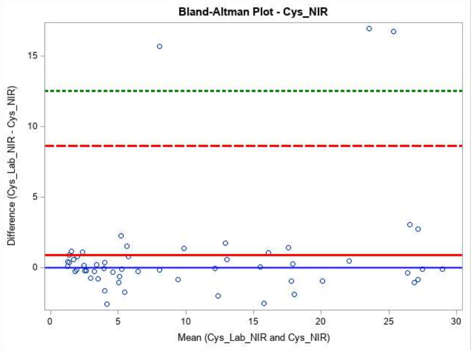

Isoleucine

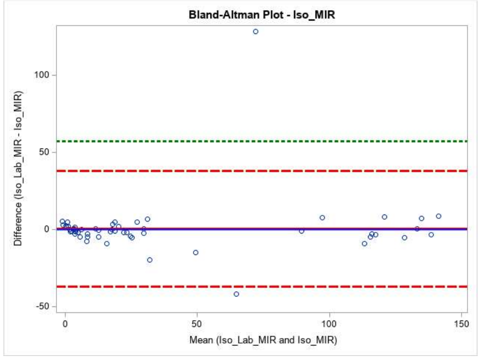

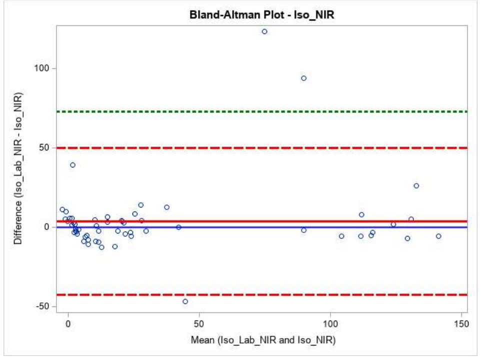

Leucine

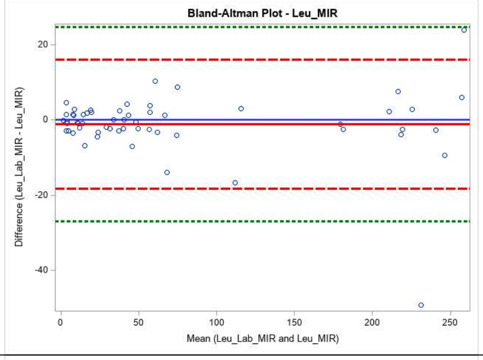

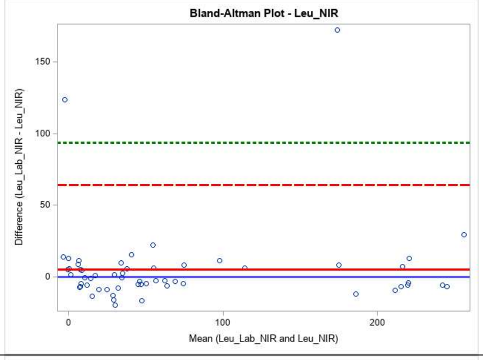

Tryptophan

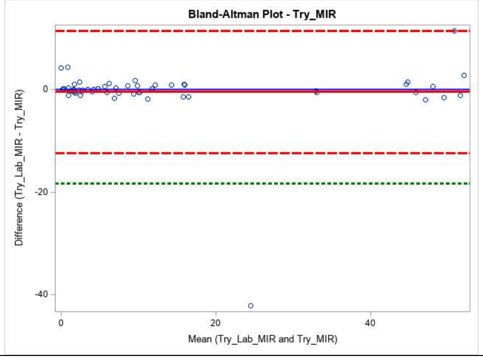

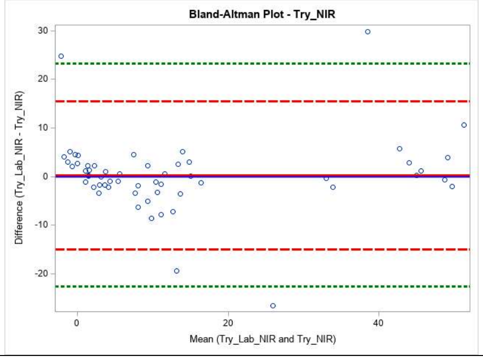

Supplement: Supplementary file 1 [file foods-15-02588-s001.zip › foods-4438212-supplementary.pdf]
